# ECG data compression using a neural network model based on multi-objective optimization

**DOI:** 10.1371/journal.pone.0182500

**Published:** 2017-10-03

**Authors:** Bo Zhang, Jiasheng Zhao, Xiao Chen, Jianhuang Wu

**Affiliations:** 1 Department of Ultrasound in Medicine, Shanghai East Hospital, Tongji University School of Medicine, Shanghai, China; 2 Shenzhen Institutes of Advanced Technology, Chinese Academy of Sciences, Shenzhen, China; 3 School of Computing and Communications, Faculty of Engineering and Information Technology, University of Technology Sydney, Sydney, Australia; Tianjin University, CHINA

## Abstract

Electrocardiogram (ECG) data analysis is of great significance to the diagnosis of cardiovascular disease. ECG compression should be processed in real time, and the data should be based on lossless compression and have high predictability. In terms of the real time aspect, short-time Fourier transformation is applied to the processing of signal wave for reducing computational time. For the lossless compression requirement, wavelet-transformation that is a coding algorithm can be used to avoid loss of data. In practice, compression is required to avoid storing redundant recording data that are not useful in the diagnosis platform. The obtained data can be preprocessed to remove noise by using wavelet transform, and then a multi-objective optimize neural network model is used to extract feature information. Compared with the existing traditional methods such as direct data processing method and transform method, our proposed compression model has self-learning ability to achieve high data compression ratio at 1:19 without losing important ECG information and compromising quality. Upon testing, we demonstrated that the proposed ECG data compression method based on multi-objective optimization neural network is effective and efficient in clinical practice.

## 1. Introduction

Electrocardiogram (ECG) is widely used in modern medicine as a diagnostic parameter. However, medical experts has to record huge chunks of such clinical data, and if these data cannot be compressed, it will increase storage cost due to large hard-disk space required. From the technical aspect, ECG data compression has these characteristics: 1) real time, lossless compression and high compression rate, and 2) the compression data can be used directly without full decompression. At the same time, electrocardiogram (ECG) that is recorded by automatic monitoring has significance to the diagnosis of cardiovascular disease. However, it usually takes a long time to record ECG data. On the other side, a large amount of electrocardiogram data is required to be analyzed and stored, while some of the meaningful feature information in these data is useful to diagnose. Therefore, it is necessary to adopt data compression algorithm to conduct compression on electrocardiogram data, in order to improve the storage and analysis efficiency of electrocardiogram.

The current ECG data compression algorithms [1, 2] can be divided into three classes: 1) direct data processing; 2) transform; and 3) neural network approaches. Direct ECG data processing method usually conducts data compression by eliminating redundant information in ECG, by using methods such as Evolutionary Computation, Turning Point Scan-Along Polygonal Approximation, and Differential-Pulse Coding Modulation (EC, TP, SAPA and DPCM) algorithms [3–5]. Transform method usually conducts data compression by mathematical function, such as Kanade Lucas Tomasi, Discrete Cosine Transform, Fast Fourier Transform (KLT, DCT and FFT) algorithms. Based on other school of thoughts, the method that is based on neural network [6–9] usually conducts data compression by extracting the feature information implied in ECG through self-learning.

ECG data compression method that based on neural networks has gained growing attention for its characteristics, which pertains to strong adaptability, parallel processing, good quality of configurable waveform, and anti-interference. On one hand, the ECG data compression should achieve a data compression ratio as high as possible; on the other hand, it is required not to lose valid information or minimize losing electrocardiogram information. Hence, a suitably designed multi-objective function can optimize ECG data compression. If the current neural network based on one objective function is applied to achieve compression, we can only get a local optimal solution due to the focus on the optimization of one objective in data quality improvement. It is worthwhile noting that the neural network can easily fall into local minimum and lose ECG data.

Therefore, this paper proposes a theory model of multi-objective optimization neural network based on multi-objective constrained optimization theory [10–13], and then it studies the ECG data compression method that based on the multi-objective optimization neural network. Generally, this method is based on the changes of ECG characteristics so that neural network can learn under the guidance of the multi-objective function and adjust its structural parameters (i.e. coupling weight and offset value). With the purpose of extracting the feature information that implied in the ECG, it can realize effective ECG data compression [14–16]. In our paper, we study the theoretical model and learning algorithm of multi-objective optimization neural networks, and then discusses ECG compression based on an optimizing neural network. Finally, we confirm the feasibility and advancement of this method through various carefully designed computational experiments in this paper.

## 2. Methodology

In this section, we present the mathematical formulations of the Discrete Wavelet Transform approach and neural network approach, which are implemented in this paper.

### 2.1 Wavelet transform used in ECG data compression

**Fig 1:**
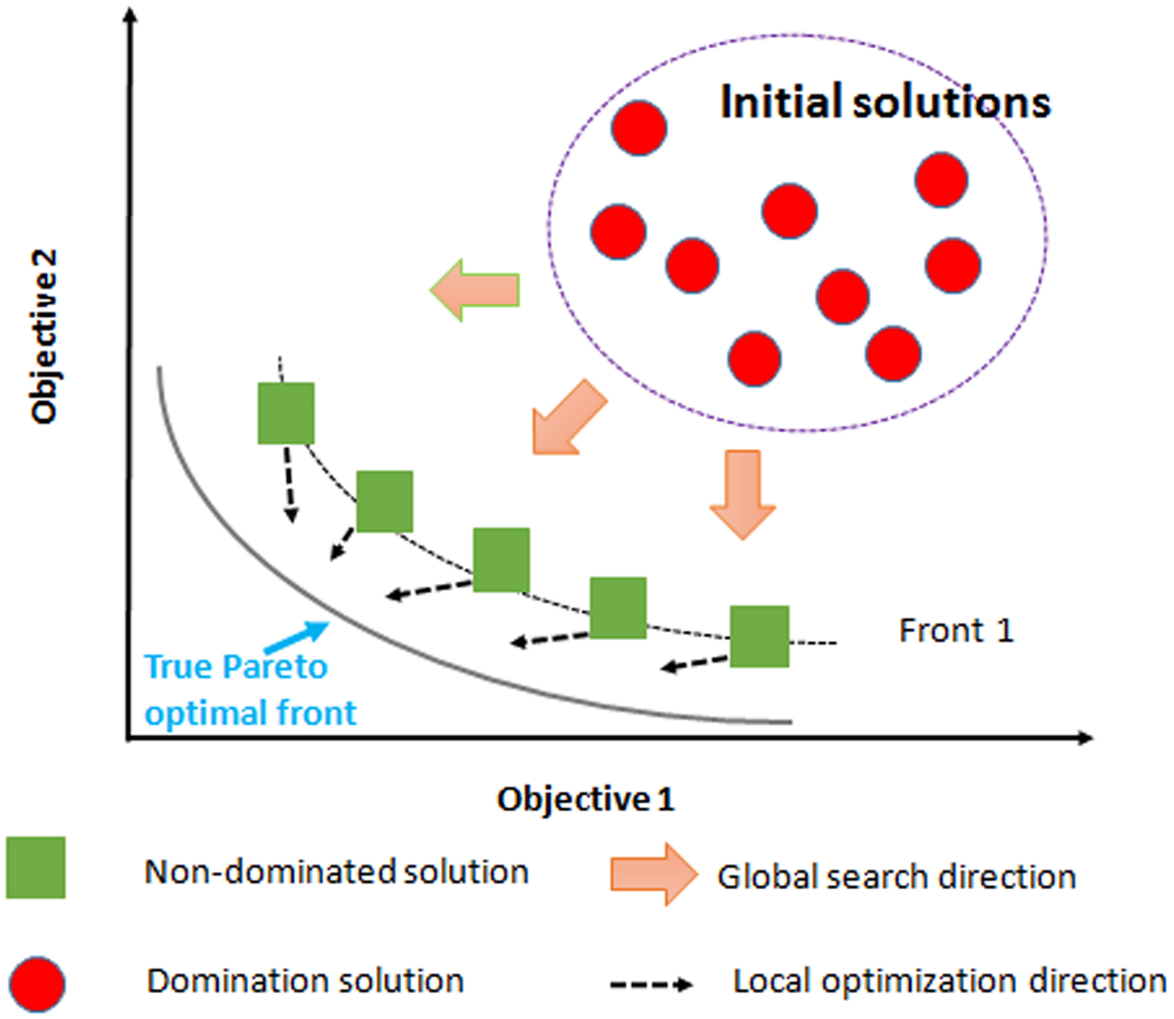
Multi-objective optimization leading to s Pareto-front of all solutions as the main objective.

ECG feature extraction [4, 17, 18] is required to remove noise signal before feature extract processing due to vulnerability from noise in the environment. Then, a Pareto-optimal solution is required to achieve the best data compression versus compromising high quality (Fig 1). Wavelet transform is greatly effective for the instantaneous and time variant signal, which can help to eliminate baseline drift noise. A wavelet transform module has an input signal, which is defined as an integration of smaller version of the mother wavelet signal. Here, we present the integral equations of our wavelet functions:

$$\begin{array}{ll} W_{s}f\left(t\right)=\frac{1}{s}\int_{\infty }^{\infty }f\left(\tau \right) & s\left(\frac{t\;\tau }{s}\right)d\tau . \end{array}$$ (1)

Wavelet scale parameters generally select a value of 2 as the form of exponent that results in the expression *s* = 2^*j*^, where *j* = 1,2,…,*m*. Note that *φ* is the parameter of the mother wave function. Eq (1) can hence be expressed as:

$$\varphi_{s}(t)=\frac{1}{s}\varphi \left(\frac{t}{s}\right).$$ (2)

Following the previous formulation, the wavelet transform can be expressed in Eq (3) as:

$$\begin{array}{ll} W_{2^{j}}f(t)=\frac{1}{\sqrt{2^{j}}}\int_{\infty }^{+\infty }f(\tau ) & \sqrt{2^{j}}\left(\frac{t\;\tau }{\sqrt{2^{j}}}\right) \end{array}d\tau .$$ (3)

**Fig 2:**
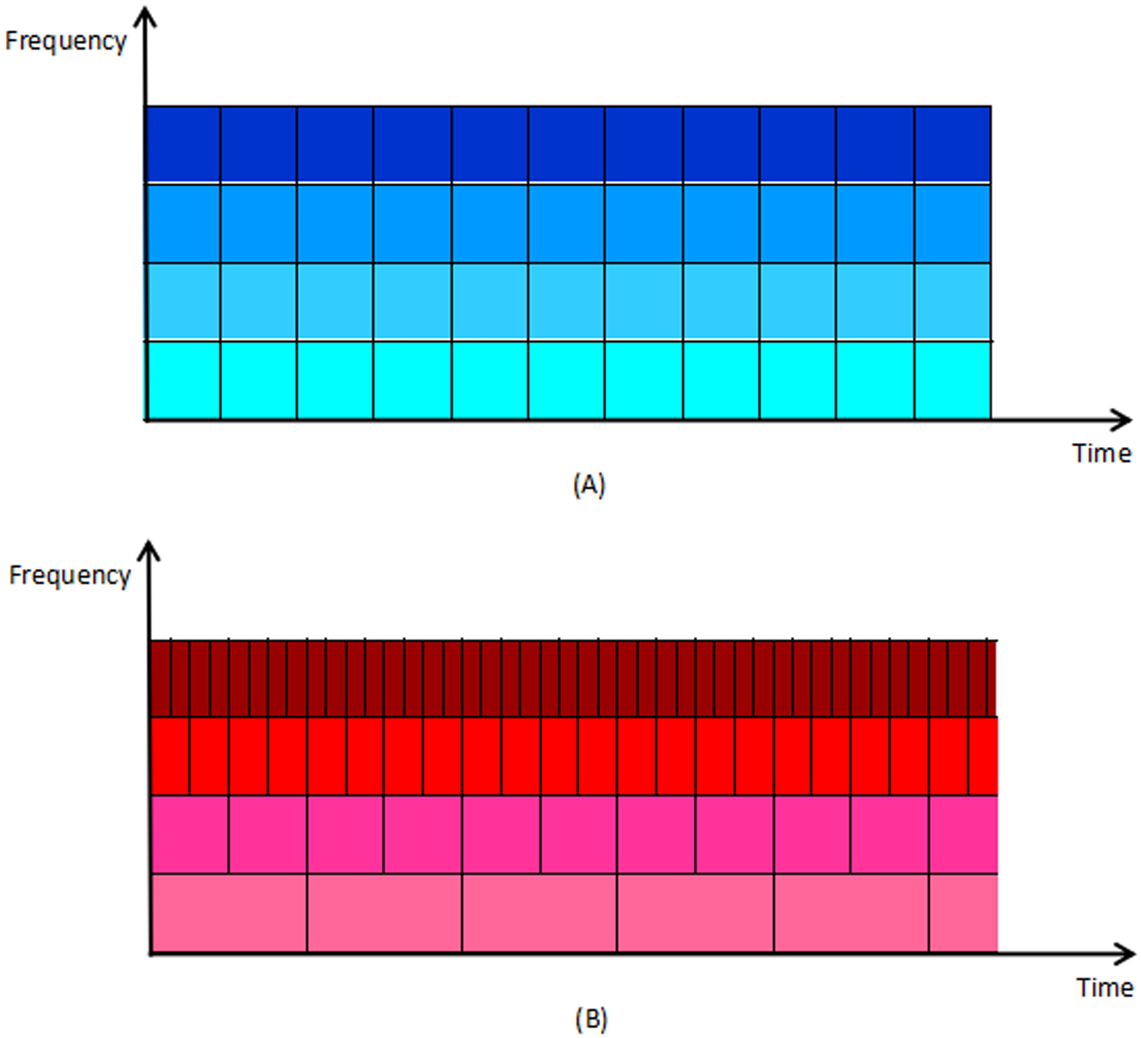
Determination of sinusoidal frequency and phase content of local sections of ECG signal versus time based on (A) short-time-Fourier-transformation; and (B) wavelet-transformation.

Discrete signal requires the use of Discrete Wavelet Transform (DWT). Now, binarization of digital signals based on the DWT algorithm can be performed to give Eqs (4) and (5) as follows:

$$W_{2^{j}}f(n)=\sum_{k\in Z}g_{k}S_{2^{j-1}}f(n-2^{j-1}k),$$ (4)

$$S_{2^{j}}f(n)=\sum_{k\in Z}h_{k}S_{2^{j}-1}f(n-2^{j-1}k),$$ (5)

where $S_{2^{j}}$ is a smooth function, $S_{2^{j}}f(n)$ is the original signal (low frequency coefficients) that serves as the approximation function, and $W_{2^{j}}f(n)$ is the original signal (high frequency coefficients). It is worthwhile noting that *h*_*k*_ and *g*_*k*_ pertain to a low-pass and high-pass filter coefficients respectively. Details of ECG signal can be observed after the DWT process. Selection of wavelet functions in the decomposition process is the key to analysis ECG signals, and hence a scale through short-time Fourier transformation and wavelet-transformation is to be used (Fig 2).

In a baseline breathing exercise, the frequency ranges from 0.15Hz to 0.3Hz. Wavelet transformation can eliminate baseline drift of signals noise disturbance, because there is no latency and reduced distortion. Wavelet ECG signal degradation for approximate signal (high amplitude and low frequency signals) and the detail signal (low amplitude of high frequency signals) can help to distinguish the desired signal and noise signal.

### 2.2 ECG testing and selection of feature

Electrocardiographic signal, which is based on the electrical activity from the heart, is made up of a series of waves including the R wave, QRS-wave, P wave, T wave, and U wave. The QRS wave represents ventricular depolarization process two potential changes and the first downward wave of QRS wave is the Q waves. Due to the R-wave arrived amplitude maximum, it is easy to detect the QRS wave after locating the position of R. QRS complex detection algorithm based on wavelet transform, the core is in a scale or search within a certain scale wavelet transform modulus maxima-minima between zero R-wave locations. [19–21] Scale wavelet transform can be achieved following these steps:

1. The *f*(*n*) of ECG can transform to $W_{2^{j}}f(n),(j\in z^{+})$, which is based on small wavelets coefficients. This process utilizes the secondary wavelets and multi-scale decomposition of samples.
2. When *j = 3*, the positive threshold *s*_1_ and negative threshold *s*_2_ can detect the maximal and minimal wavelets.
3. Locate the value that is over zero point, between the maximum value and minimum value.
4. The modified point of R-wave location can be acquired by $\frac{2^{3-1}}{2}=4$.

After locating R, we can be certain that every beat will contain the P-QRS-T waves. Note that our ECG database is based on 251 points in a heartbeat cluster, such that we have R before 90 points, and R behind 160 points as two groups. The QRS-wave signal frequency content concentrate on details with a scale of 3, 4, and 5. Next, the T and P waves mainly concentrate details with a scale of 3, while other levels that do not contain noise are discarded. Notably, the time domain characteristics in ECG and RR intervals constitute a feature vector, which forms the foundation of signal classification.

### 2.3 Model of multi-objective optimization neural network

**Fig 3:**
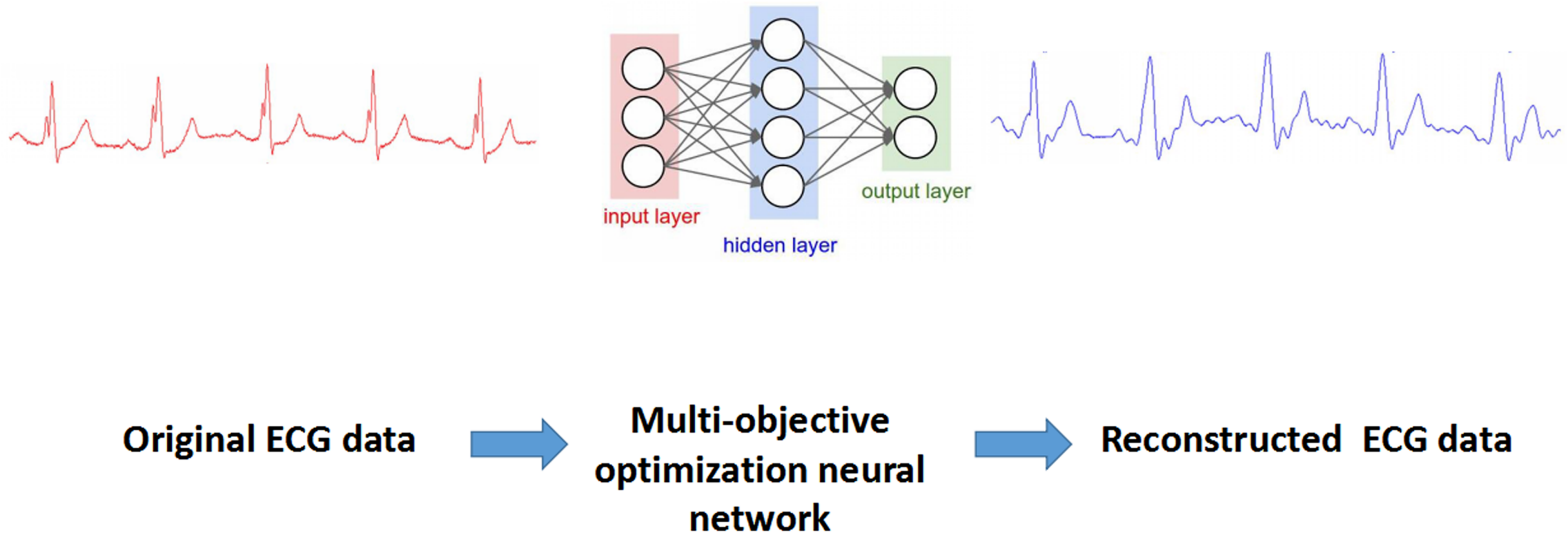
Flowchart of multi-objective optimization neural network for reconstruction of ECG data.

Neural network has appeared in increasing applications in the field of optimal computation, pattern recognition, intelligent control, and signal processing. However, multi-objective function [22, 23] is an index in a large number of engineering applications. Generally speaking, the feature of ECG can be reserved in this pattern, and it has high access ability without losing any useful information through the NN hidden layers (Fig 3). Therefore, the simultaneous optimization of multi-objective function shall be described through the following mathematical problem, and the integral in Eq (6) can be expressed as:

$$\text{min}\vec{y}=\underset{\vec{x}\in D}{\vec{f}(\vec{x})},$$

$$\text{min}\vec{y}=(f_{1}(\vec{x}),f_{2}(\vec{x}),\ldots ,f_{m}(\vec{x})),$$ (6)

Where $f(\vec{x})=(f_{1}(\vec{x}),f_{2}(\vec{x}),\cdots ,f_{m}(\vec{x}))$ is the multi-objective vector criterion function, $\vec{x}$ is an Euclidean space vector of *n*-dimensional, *X* is a set of constraints, $\vec{y}$ is the objective vector, and *D* is search space.

In a multi-objective optimization problem, the non-inferior solution concept is usually adopted to describe the solution of vector function optimization. This means that a feasible decision vector *x*’ ∈ *X* is non-inferior solution, and *x*’ ∈*X* does not exist, and therefore Eq (7) becomes:

f(x)≤f(x′). (7)

The non-inferior solution of multi-objective optimization problem can be obtained by the following, and then Eq (8) can be expressed as:

$$\underset{\vec{x}\in X}{\text{min}}\sum_{i=1}^{m}\omega_{i}\cdot f_{i}(x),$$ (8)

where *ω*_*i*_ > 0 and $\sum_{i=1}^{m}\omega_{i}=1$.

In the case of a convex objective function and convex constraint, *x* is completely determined by the changes of ${\vec{\omega }}_{i}=(\omega_{i1},\omega_{i2},\cdots \omega_{im})$, so that a multi-objective convex optimization can be solved by weighting and secularization optimization. If a feed forward neural network is used to solve the multi-objective optimization problem, then this neural network can minimize the energy function in the following form, and the integral in Eq (9) can be expressed as:

$$E=\sum_{i=1}^{m}\omega_{i}\cdot f_{i}(x).$$ (9)

According to Eq (9), the learning equation for multi-objective optimization is derived as follows, and then Eq (10) becomes:

dωijdt=−α∑k=1mωk∂fk(x)∂ωij, (10)

where *ω*_*ij*_ is a weight between neurons *i* and neurons *j*, *α* is the neural network learning rate, and *f*_*k*_(*x*), (*k* = 1,2,⋯*m*) is the objective function that is to be determined by the existing problem. Finally, Eq (11) becomes:

$$\begin{array}{lll} \omega_{i}>0 & \text{and} & \sum_{i=1}^{m}\omega =1. \end{array}$$ (11)

### 2.4 ECG data compression based on multi-objective optimization

**Fig 4:**
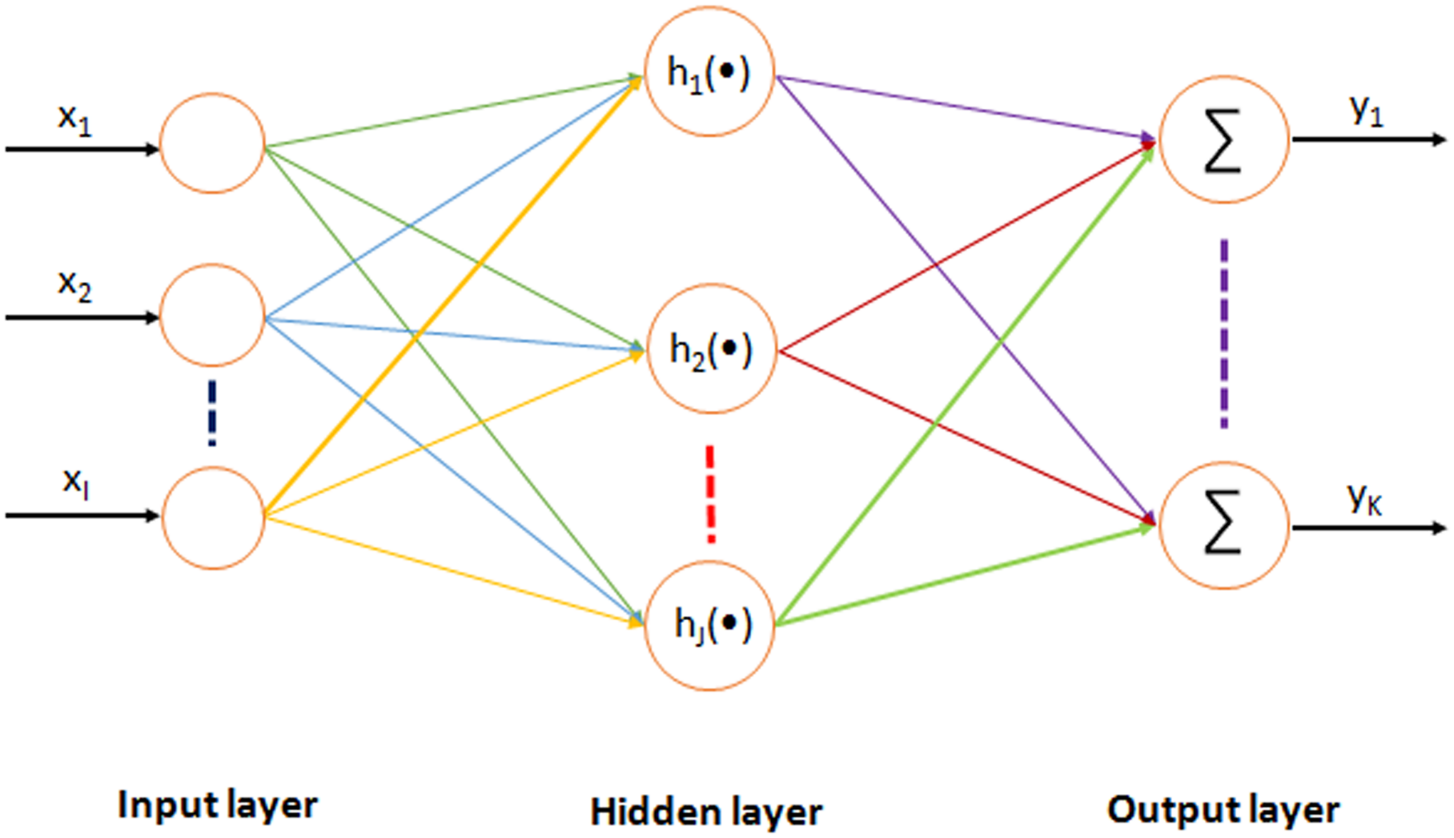
Neural network connections with the input, hidden and output layers of nodes representing a connection from a neural output to the input of a neuron.

The structure of the multi-objective optimization neural network of ECG data compression is shown in Fig 4. It is a three-layer feed forward neural network, including input layer, implication layer and output layer. The input of neurons in input layer is the sampling point data of ECG. The neurons in hidden layer change according to the characteristics of ECG by learning to adjust the weight and bias value between it and input layer neurons. It is possible to extract the feature information implied in ECG (expressed as the output information of implied neuron). [15, 24, 25] ECG waveform after data compression can be reconstructed by output layer neurons based on the ECG feature information that is extracted by hidden neurons, based on its weight and offset value with hidden layer neurons. If the general back propagation (BP) algorithm is applied to train the above-mentioned neural network, then the following problems exist: 1) there is a long processing time during network training; 2) the solution easily falls into a local minimum; and 3) the weights of the hidden layer neurons is difficult to determine.

The hidden layer decides the compression ratio. Note that if there are too many hidden layer neurons, the data compression ratio will decrease. On the other hand, if the hidden layer neurons are too few, the data compression performance will reduce, and resulting in significant distortion of reconstructed ECG. Nevertheless, an effective and practical ECG data compression algorithm requires not only a high data compression ratio but also, the reconstructed ECG shall retain or minimize loss of the effective ECG information as much as possible. Meanwhile, the real-time performance of algorithm is also required in practice [10, 26–29]. Therefore, ECG data compression can be expressed as a multi-objective optimization problem mathematically, which means to seek for the optimal data compression effect under the constraints of following multi-objective functions: 1) data compression ratio; 2) valid information loss after data compression; and 3) the real-time performance of data compression.

The model of multi-objective neural network that is discussed in the previous section is presented here to solve multi-objective optimization problem. Following that, it can be used to achieve multi-objective compression of ECG data. Currently, the key question is how to summarize the multi-objective optimization function of ECG data compression. [30, 31]

At present, the percentage root-mean-squared difference (PRD) and correlation coefficient (CC) are widely adopted as indicators to evaluate the loss of effective information after data compression, then Eqs (12) and (13) becomes:

$$PRD=\frac{\sqrt{\frac{1}{N}\sum_{i=1}^{N}\left(\left(r_{i}-\bar{r}\right)-{\left(o_{i}-\bar{o}\right)}^{2}\right)}}{\sqrt{\frac{1}{N}\sum_{i=1}^{N}{\left(o_{i}-\bar{o}\right)}^{2}}},$$ (12)

$$CC=\frac{\frac{1}{N}\sum_{i=1}^{N}\left((o_{i}-\bar{o})\cdot (r_{i}-\bar{r})\right)}{\sqrt{\frac{1}{N}\sum_{i=1}^{N}\left(o_{i}-\bar{o}\right)}\cdot \sqrt{\frac{1}{N}\sum_{i=1}^{N}{\left(r_{i}-\bar{r}\right)}^{2}}},$$ (13)

Where *o*_*i*_ indicates the value of sampling point *i* in the original waveform, *r*_*i*_ is the value of sampling point *i* in the restored waveform, to restore the value of the sampling point of the waveform of *i*, $\bar{o}$ is the average value of all sampling points in the original waveform, and $\bar{r}$ is the average value of all sampling points in the restored waveform. [32] From Eqs (12) and (13), it can be seen that PRD represents the error magnitude contained in the waveform; and CC represents the correctness of restored waveform. Therefore, multi-objective optimization function of ECG data compression can be summarized as follows in Eq (14):

$$E=W_{1}\cdot N_{d}+W_{2}\cdot PRD+W_{3}\cdot (1-CC),$$ (14)

where *N*_*d*_ represents the number of neurons in hidden layer, PRD represents normalized RMS error, and CC represents the correlation coefficient. In addition, the *W*_1_, *W*_2_ and *W*_3_ represent the following indicators: 1) the weight of compression ratio, 2) normalized RMS error, and 3) correlation coefficient in the multi-objective ECG data compression, respectively.

The implication is as follows: solving the minimum value of Eq (14) requires seeking the optimal compromise solution between the effective ECG information and the data compression ratio to be as high as possible. The main purpose of the first term in Eq (14) is to improve the data compression ratio while the second term mainly reflects the error magnitude contained in ECG waveform, with the purpose to reduce the total amount of error in the restored electrocardiogram [1, 5, 20, 33]. The third term mainly reflects the correctness of ECG waveform restoration, with the purpose to reduce the recovery error of all sampling points in ECG waveform. According to [32–34], we can derive the learning equation of neural network for ECG data compression in Eq (15) as:

$$\begin{array}{ll} \frac{d\omega_{ij}}{dt}= & \alpha \frac{\partial E}{\partial \omega_{ij}}, \end{array}$$ (15)

which then leads to Eq (16) as

$$\frac{d\omega_{ij}}{dt}=\alpha \left(\omega_{1}\frac{\partial N_{d}}{\partial \omega_{ij}}+\omega_{2}\frac{\partial PRD}{\partial \omega_{ij}}+\omega_{3}\left(\frac{\partial CC}{\partial \omega_{ij}}\right)\right),$$ (16)

Where *α* is the neural network learning rate. Note that *ω*_1_, *ω*_2_ and *ω*_3_ are positive numbers less than 1, and that their sum of weights is such that *ω*_1_+*ω*_2_+*ω*_3_ = 1.

## 3. Results and discussion

### 3.1 Experimental verification

In order to verify the effectiveness and advancement of theoretical model and learning algorithm of multi-objective optimization neural network in the applications of ECG data compression, we conduct ECG data compression study based on neural network with partial ECG waveform T100, T105, T106, T108, T111, T112, T217, T219, T220 and T221 from MIT / BIH ECG database [2, 3, 14].

In our experiment, all parameters are set as follows. For the ECG waveform of data compression, each heart beat consists of 105 points before the R-wave peak, and165 points after the R-wave peak. Then, we conduct samples in the 270 points data such that all 15 points are used around the R point. For the other sections, samples are carried out every 6 points, so that each heart beat has 70 data points.

### 3.2 Reconstructed waveform based on other ECG data compression algorithms

The neurons number in both input layer and output layer is 70, and the neurons number in hidden layer can be obtained by a multi-objective optimization function. The inputs neurons in the neural network correspond to the sampling data of ECG waveform. The outputs of hidden neurons correspond to the implicit feature information of each ECG waveform. Next, after compression, the ECG waveform data is acquired through the weight between input neurons and hidden neurons. The sampling data of input ECG waveform and output neurons corresponds to the data of reconstructed ECG waveform, which is acquired using the weight between hidden neurons, output neurons, and the output of hidden neuron. At this time, the weight between input neurons and hidden neurons, the weight between hidden neurons and output neurons, and the offset value between hidden neurons and output neurons are obtained by neural network through learning in regards of ECG data compression. (Fig 4) First of all, 14 neurons are selected as hidden neurons, and we select 40 waveforms from the T100 ~ T221 series in order to train the neural network for 10,000 cycles. Here, the E value in multi-objective compression function of ECG data is 3.557, (parameters in the learning algorithm of multi-objective optimization neural network are set as: *ω*_1_ = 0.25, *ω*_2_ = 0.45, *ω*_3_ = 0.3, and *α* = 0.4). Then, we implemented 16 neurons as hidden neurons, where parameters of neural network training data and training time are the same as above. Here, the E value in multi-objective compression function of ECG data is 3.975. Finally, 12 neurons are selected as hidden neurons, whose training data, training time and learning algorithm parameters are the same as above. Then, the *E* value in multi-objective compression function of ECG data is 3.764.

**Fig 5:**
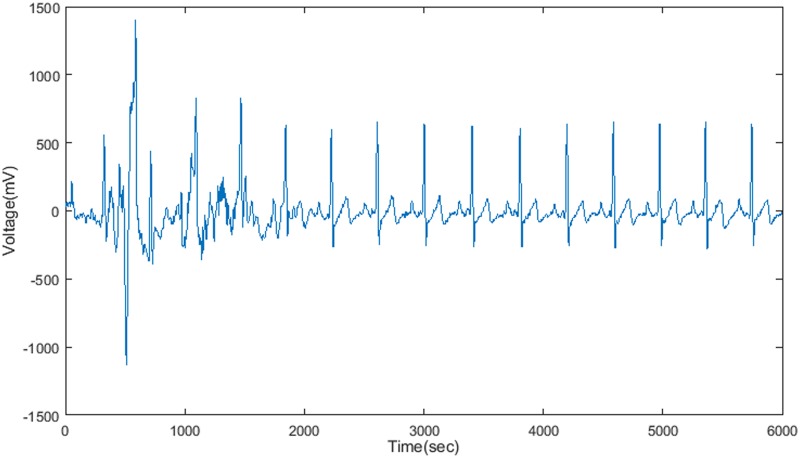
Reconstructed signal output after ECG compression for a longer period of 6000 seconds.

As can be seen from the above results, selecting 14 neurons as hidden neuron is appropriate. Moreover, in order to check the learning outcomes of neural network, the studied and non-studied ECG waveforms (based on selection of 40 waveforms from T100 ~ T221 ECG waveform, from which 67% are studied) are regarded as the input of neural network, then the hidden layer neurons record the compressed data of each ECG waveform. In the following modules, the output layer neuron can reconstruct the ECG waveform based on the output information of hidden neurons, and the weight between hidden neurons and output neurons that obtained through neural network learning. At this time, the evaluation indexes values of ECG compression are: data compression ratio is 1:19, PRD = 12%, and CC = 99%, as shown in Fig 5.

**Fig 6:**
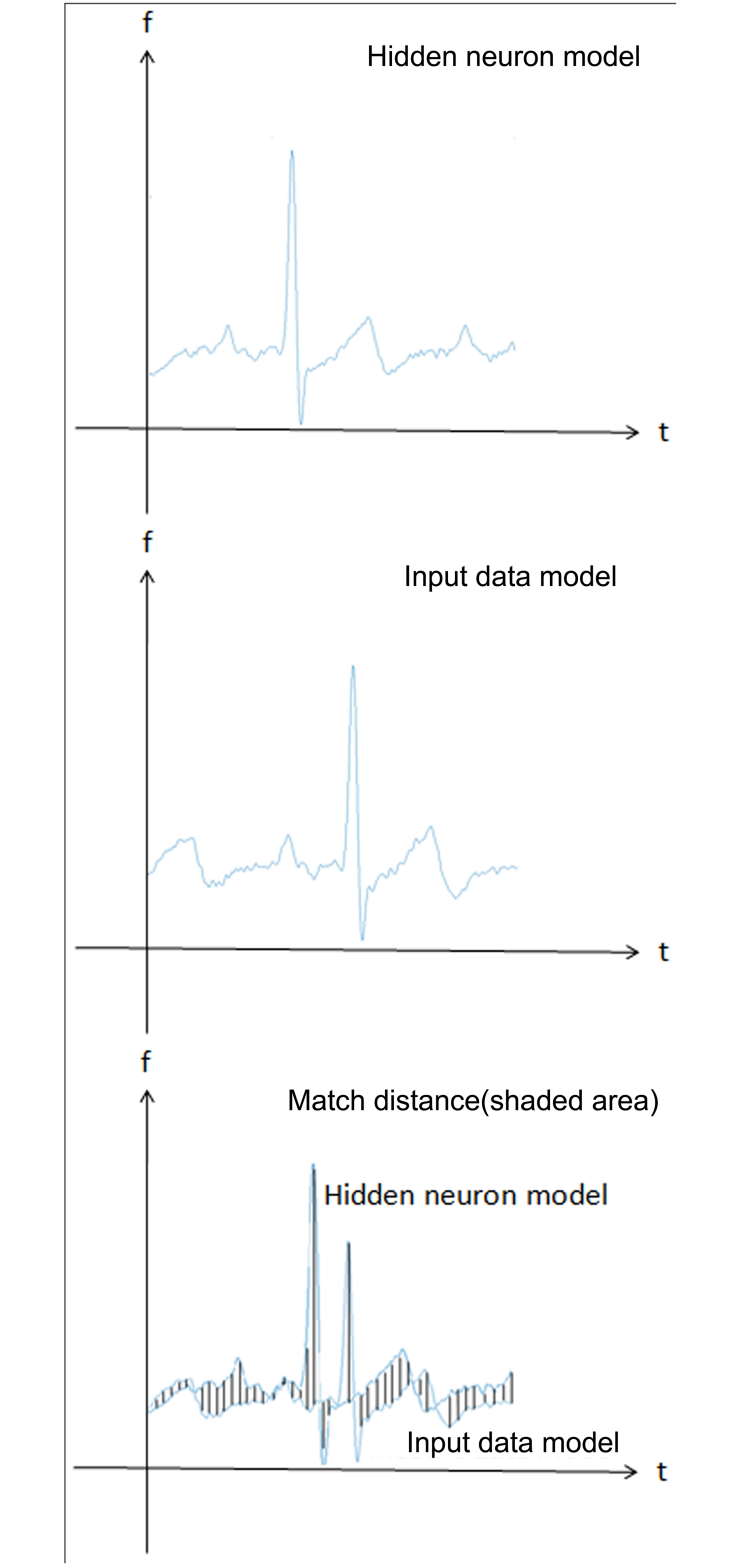
Comparison of waveform by the hidden neuron and input data models using a match distance approach.

The hidden neurons match the output neurons based on Dynamic Time Warping (DTW). Notably, the DTW can recognize the all ECG waves, and then classify different ECG waves into output layers. The frequency of the wave can be detected by trained waves, and the trained wave can predict the income waves according to an Euclidean metric. There are *M* frames in hidden neuron and *N* frames in input neuron, where *d* represents the distance between the hidden neuron and input neuron. Each frames based on *M* and *N* has a certain distance (Fig 6). Therefore, the output data can be screened by this distance. The defined distance is set as 0.3 in order to raise the accurate of output data.

Using the above analysis, it can be seen that multi-objective optimization neural network that used for ECG data compression is significantly better than several other data compression algorithms. Neural networks can adjust the parameters of network structure by learning the ECG data compression that comes with different characteristics. In addition, it can carry out learning under the guidance of multi-objective function in order to achieve the best result of data compression. This means that we are able to achieve a data compression ratio to be as high as possible without losing useful ECG information or losing as few information as much as possible.

**Fig 7:**
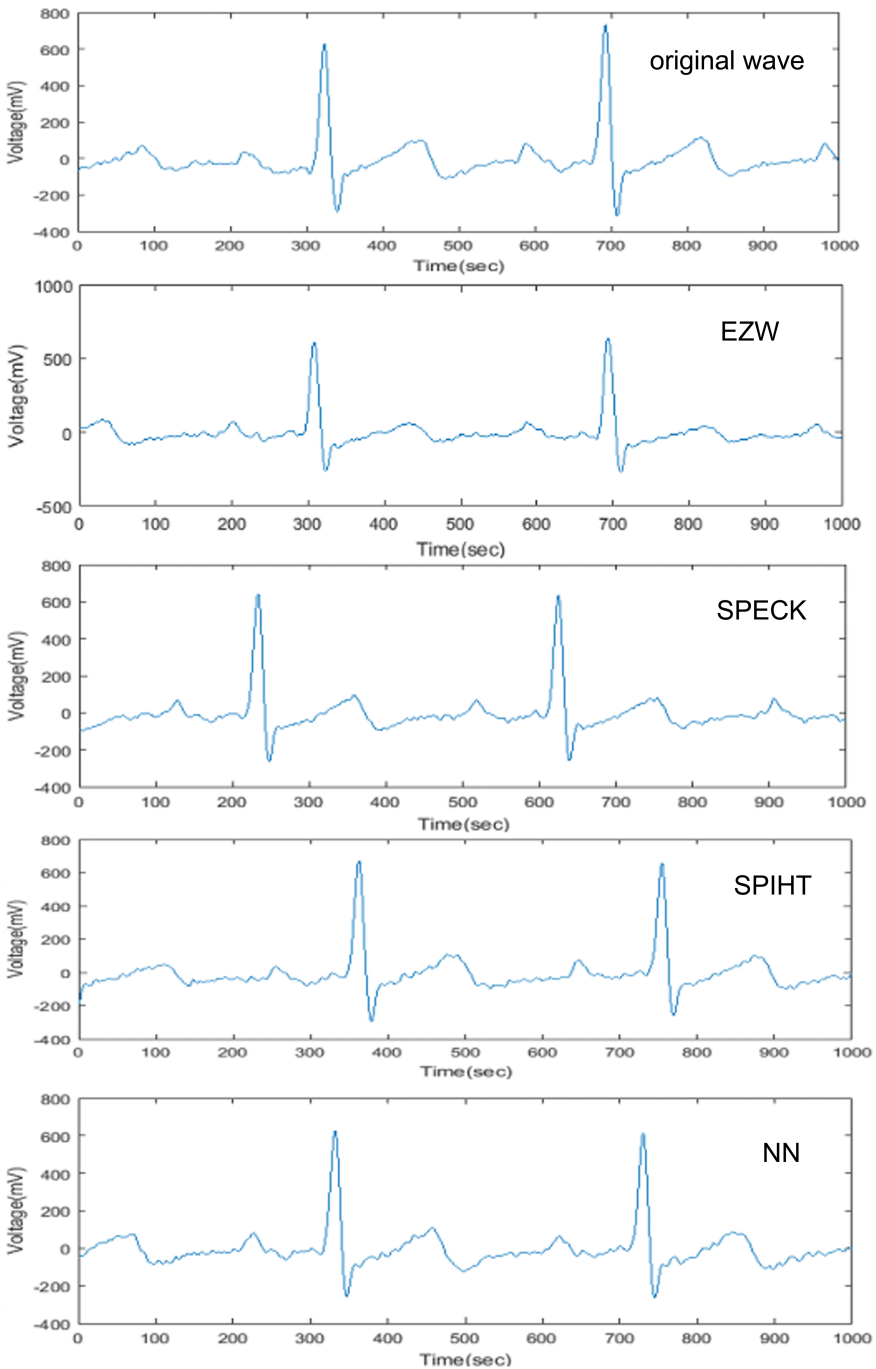
Reconstructed ECG signal waveform based on the wavelet compression and neural network methods in comparison with the original signal.

**Fig 8:**
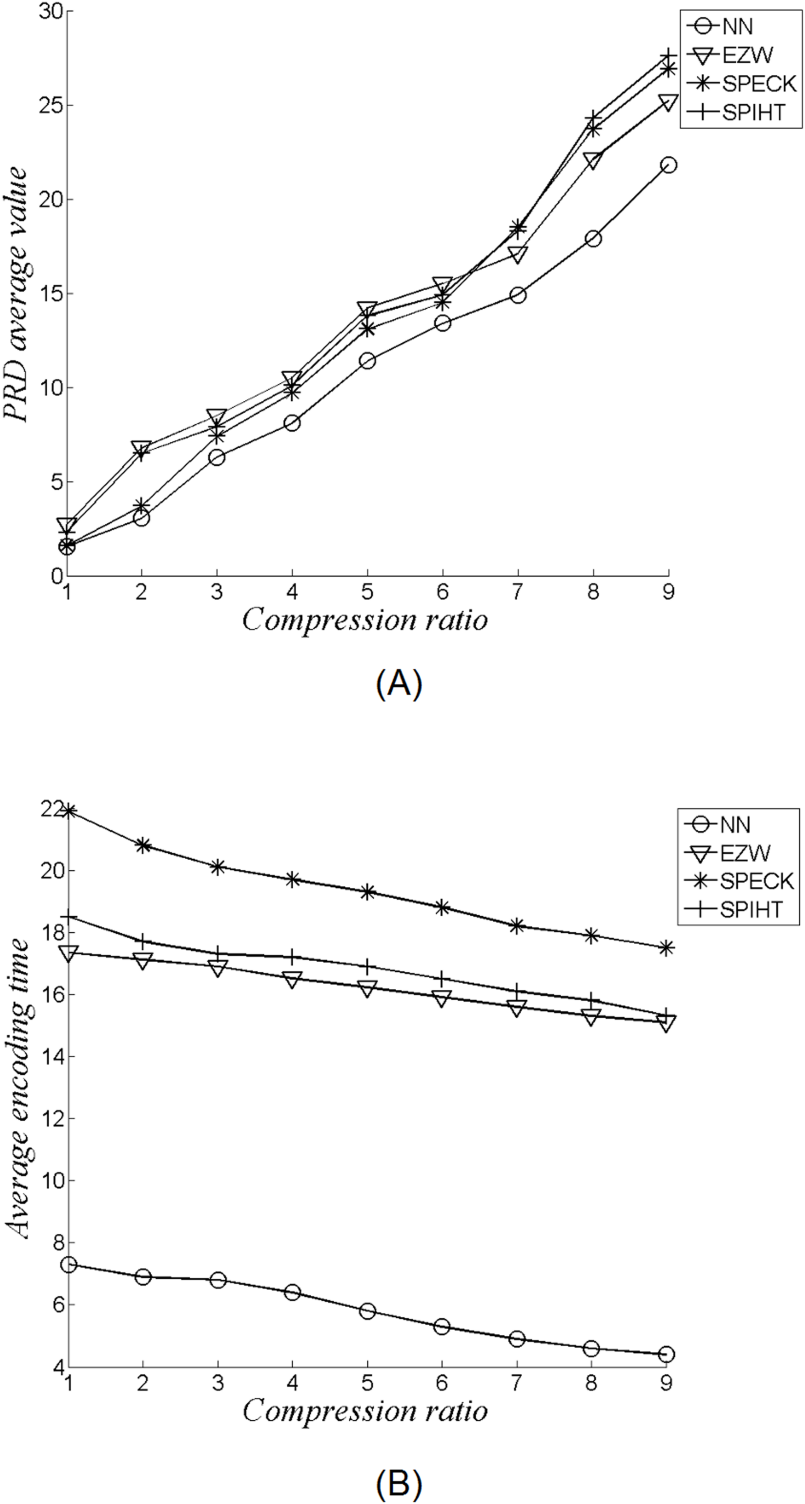
Average percentage root-mean-squared difference (PRD) results based on different ECG data compression ratios using transform and neural network approaches (A); Average encoding time versus ECG compression ratio using transform and neural network approaches (B).

Fig 7 demonstrates the reconstructed ECG signals after undergoing the data compression process. Our method based on neural network can achieve fewer probe average values in different compression ratio in comparison to other methods such as Embedded Zerotree Wavelet (EZW), Set Partitioning Embedded Block Coder (SPECK), and Set Partitioning Hierarchical Trees (SPIHT) as demonstrated by Fig 8(A). At same time, our method requires less computational time as compared to these methods based on the same compression ratio. (Fig 8(B))

## 4. Conclusion

In this paper, we put forward our mathematical model and learning algorithm for a neural network that is based on multi-objective optimization. This approach is then successfully applied onto ECG data compression. In our computational experiments, a satisfactory ECG data compression result is achieved, and we compared our neural network approach with the wavelet transform approaches to demonstrate its superiority. For future implementation, it may be of interest to compare this type of technique with direct data processing methods. Our model can process the useful data adaptively and efficiently, which comes at a lower cost in comparison with the traditional ECG compression techniques already in practice. Furthermore, the effectiveness and advancement of ECG data compression method that based on multi-objective optimization neural network are confirmed through comparison with these existing techniques.

## Abbreviations

BP: Back Propagation
CC: Correlation Coefficient
DCT: Discrete Cosine Transform
DPCM: Differential-Pulse Coding Modulation
DTW: Dynamic Time Warping
DWT: Discrete Wavelet Transform
EC: Evolutionary Computation
ECG: Electrocardiogram
EZW: Embedded Zerotree Wavelet
FFT: Fast Fourier Transform
KLT: Kanade Lucas Tomasi
PRD: Percentage Root-mean-squared Difference
SAPA: Scan-Along Polygonal Approximation
SPECK: Set Partitioning Embedded Block Coder
SPIHT: Set Partitioning Hierarchical Trees
TP: Turning Point
